# Prognostic impact of p16 and p21 on gastroenteropancreatic neuroendocrine tumors

**DOI:** 10.3892/ol.2013.1610

**Published:** 2013-10-09

**Authors:** SHUZHENG LIU, YUXI CHANG, JIE MA, XU LI, XIAOHONG LI, JINHU FAN, RONG HUANG, GUANGCAI DUAN, XIBIN SUN

**Affiliations:** 1 Department of Epidemiology, College of Public Health of Zhengzhou University, Zhengzhou, Henan 450001, P.R. China; 2 Henan Cancer Research and Control Office, Henan Cancer Hospital, Zhengzhou, Henan 450008, P.R. China; 3 Department of Pathology, The Affiliated Tumor Hospital of Zhengzhou University, Henan Cancer Hospital, Zhengzhou, Henan 450008, P.R. China; 4 Department of Cancer Epidemiology, Cancer Institute/Hospital, Chinese Academy of Medical Sciences, Beijing 100021, P.R. China

**Keywords:** gastroenteropancreatic neuroendocrine tumor, p16, p21, immunohistochemistry

## Abstract

Aberrant expression of the cell cycle kinase inhibitors, p16 and p21, has been associated with poor prognosis in a number of human malignancies. These proteins may also be involved in the development and progression of gastroenteropancreatic neuroendocrine tumors (GEP-NETs). The present study aimed to investigate protein levels of p16 and p21 in GEP-NETs and to evaluate their clinical significance. p16 and p21 protein expression was tested immunohistochemically in the tissue samples of 68 GEP-NETs. The association between expression and clinicopathological characteristics and overall survival was assessed. Low expression of p16 (no positive nuclear staining) was found in 37 (54%) cases and high p21 expression (≥5% positive nuclear staining) was detected in 23 (34%) cases. Low p16 protein levels indicated a poorer prognosis for patients graded as G2 subgroup in the univariate analysis (relative risk, 4.4; 95% CI, 1.8–10.6). No significant correlation was found between the expression of p21 and any of the clinicopathological variables. The present study indicates a prognostic relevance for p16 immunoreactivity. Low levels of p16 protein were associated with a shorter survival in the G2 subgroup of GEP-NETs. p21 protein expression was not identified to be useful as a predictive indicator in GEP-NETs.

## Introduction

Gastroenteropancreatic neuroendocrine tumors (GEP-NETs) are complicated and rare tumors arising from the neuroendocrine system of the gut. The estimated annual incidence is 1–4 cases per 100,000 individuals and recent studies have revealed an increasing incidence at several sites of the tumor (1–5). GEP-NETs are traditionally classified according to their origin from divisions of the gut (6). However, the biological and clinical characteristics of the tumors vary greatly between the subgroups. The 2010 WHO classification of endocrine tumors of the gastroenteropancreatic tract has been used more recently (7). According to the classification, GEP-NETs are graded between highly differentiated (G1) and poorly differentiated (G3). Intermediate grade (G2) GEP-NETs have a moderately aggressive, but less predictable course, while tumors of G1 grade are relatively indolent and those of G3 grade are extremely aggressive (8). Therefore, there remains a requirement for new prognostic and predictive factors in order to optimize treatments among the patients of the intermediate grade.

Immunohistochemical markers have been used in a number of tumors as effective predictors of malignant behavior. p16 and p21, the cyclin-dependent kinase inhibitors (CKIs), block cellular proliferation and govern the G1/S cell cycle checkpoint (9). These CKIs bind to cyclin dependent kinases (CDK4 and/or CDK2) and thereby prevent activation of the CDKs. Phosphorylation of the retinoblastoma protein, a key step for cell cycle progression from G1 to S phase, does not occur in the absence of activated CDKs (9). p16 and p21 expression has been studied in a number of human tumors (10–13); however, there are limited data on the expression of p16 and p21 in GEP-NETs.

To further elucidate the molecular pathogenesis of GEP-NETs and identify immunohistochemical markers for the determination of patient outcomes, the expression of p16 and p21 in a series of patients with GEP-NETs was tested.

## Materials and methods

### Patients

A total of 68 patients with GEP-NETs, undergoing surgery at Henan Cancer Hospital (Zhengzhou, China) between 2000 and 2010, were investigated in this study. The median age at diagnosis was 59 years (range, 28–86 years old). The patients were not treated by chemotherapy or radiotherapy prior to surgery. All patients were followed until mortality or until November 30, 2012. No case was lost during follow-up and the patients were censored following five follow-up years. The study was approved by the ethics committee of Henan Cancer Hospital (Zhengzhou, China). Written informed consent was obtained from the patients.

Ki-67 was restained and recounted to calculate the Ki-67 index. The Ki-67 index was determined by assessing the percentage of positively stained tumor cell nuclei. These evaluations were made in the most viable areas of the stained tumor sections that also demonstrated the highest degree of nuclear labeling According to the standards of the 2010 WHO Classification (7), tumors with a Ki-67 index of <2% were categorized as G1, 3–20% were categorized as G2 and ≥20% as G3. In this study, 9 (13.2%) patients were G1, 37 (54.4%) were G2 and 22 (32.4%) were G3.

### Immunohistochemistry

Sections (5-μm thick) were cut and deparaffinized with xylene and dehydrated in graded alcohols. Endogenous peroxidase activity was blocked with 3% hydrogen peroxide in methanol for 10 min. The sections were then treated with microwave radiation for 10 min for antigen retrieval and to block intrinsic antibody binding and then reacted with normal serum (mouse IgG) for 10 min at room temperature. The sections were subsequently incubated with primary antibodies against p16 (clone 6H12), Ki67 (clone MIB-1) and p21 (clone DCS-60.2; Maixin Bio, Fuzhou, China) overnight at 4°C, with appropriate negative and positive controls, and reacted with the horseradish peroxidase-polymer anti-mouse antibody (Maixin Bio) for 40 min. Diaminobenzidine tetrahydrochloride was used as the final chromogen. Sections were counterstained with Mayer’s hematoxylin and dehydrated in graded alcohols before mounting.

Four classes were used to score nuclear positively stained tumor cells; none, <5, 5–50 and >50% of the cells. Protein levels were classified as high for p16 when any nuclear staining was identified in the tumor tissue and for p21 when ≥5% of the tumor cells were positive, as outlined in previous studies (14,15). All slides were evaluated the same day by two pathologists to minimize the variability of the results.

### Statistical analyses

The associations between p16 and p21 protein expression and clinicopathological variables were evaluated by Fisher’s exact test. Survival rates were estimated by the Kaplan-Meier method, starting from the time of diagnosis. Prognostic analysis of p16 and p21 in GEP-NETs was performed by Cox proportional hazards regression model. When analyzing the G2 subgroup, the colon and rectum were combined with the pancreas into one group. p16 and p21 were always kept in the model and the inclusion criteria of other variables were 0.10 on forward stepwise regression. All statistical analyses were performed using SPSS software (version 11.0; SPSS, Inc., Chicago, IL, USA). P<0.05 was considered to indicate a statistically significant difference.

## Results

The immunohistochemical results in GEP-NETs are summarized in Table I. For p16, low (no positive nuclei) and high (any positive nuclei) protein levels were detected in 37 (54%) and 31 (46%) cases, respectively. Low immunoreactivity (<5% positive nuclei) of p21 was found in 45 (66%) cases and high expression (≥5% positive nuclei) was observed in 23 (34%) of the cases.

**Figure 1:**
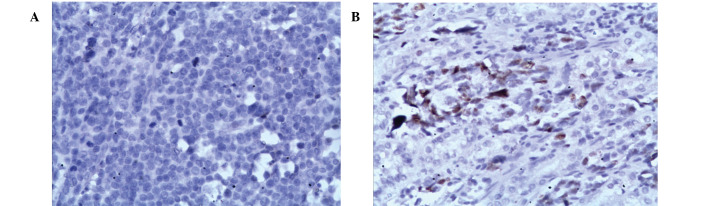
Immunohistochemical analysis showing (A) low p16 and (B) high p21 protein expression (H&E; original magnification, ×200).

No significant correlation was found between the expression of p16 and p21 and clinicopathological variables, including tumor origin, the classification, tumor size, functional status, metastasis and localization of the metastases (Table II). Examples of immunohistochemical staining for low p16 (no positive nuclei staining) and high p21 (≥5% positive nuclei staining) are presented in Fig. 1.

The associations between clinicopathological and immunohistochemical data and survival, in univariate and multivariate analyses, are presented in Table III. The univariate analysis in all cases showed a relative risk (RR) of succumbing to GEP-NETs of 1.4 (95% CI, 0.8–2.4; P=0.25) for low expression of p16 and an RR of 0.7 (95% CI, 0.4–1.2; P=0.16) for high expression of p21. In multivariate analyses, WHO classification (P<0.01) and metastasis (P=0.01) were the only parameters with statistical significance.

**Figure 2:**
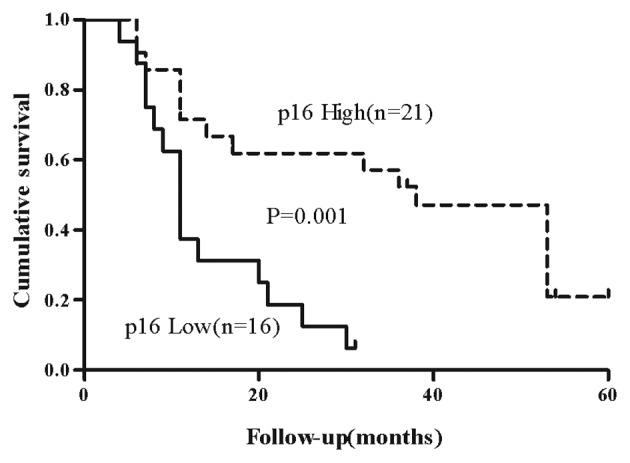
Kaplan-Meier survival curves for patients with GEP-NETs graded as G2 for high and low expression of p16. GEP-NETs, gastroenteropancreatic neuroendocrine tumors.

The results for p16 from the univariate analysis for the patients classified as G2 are depicted in Fig. 2. In the survival analysis of the cases classified as G2, low p16 indicated prognostic relevance with an RR of 4.4 (95% CI, 1.8–10.6; P=0.001), revealing a poorer prognosis for patients presenting with tumors expressing low levels of p16 (Table IV).

## Discussion

The diagnostic and prognostic role of p16 and p21 in human tumors has been evaluated for a number of years. However, there are few studies regarding the expression status of p16 and p21 in GEP-NETs. In this study, 68 patients with GEP-NETs were analyzed and p16 was found to represent a valuable prognostic marker for survival.

The p16^ink4a^ tumor suppressor protein is encoded by the CDKN2A gene and functions as an inhibitor of CDK4 and CDK6. Inactivation of the CDKN2A gene contributes to the bypass of a mid-late G1 restriction point (R point) and is associated with progression to malignant disease. Inactivation of the p16^ink4a^ gene by deletion, methylation and point mutation has been found in ~50% of all human tumors (16–19). In GEP-NETs, inactivation of the CDKN2A gene appears to confer a more malignant prognosis (20).

However, loss or reduction of p16^ink4a^ transcription or staining without marked inactivation of the CDKN2A gene has also been reported (21). Arnold *et al* found that p16 expression was lost in 49/118 (42%) of GEP-NETs and there were no promoter methylation of the gene (14). Therefore, an additional mechanism may contribute to GEP-NETs that retain the wild-type CDKN2A gene, potentially underestimating the percentage of p16^ink4a^ inactivation.

In the present study, p16 expression was lost in 37/68 (54%) of cases. Among the G2 subgroup, a low level of p16 expression was shown to be associated with decreased overall survival.

The cyclin-dependent kinase inhibitor p21 (Waf1/Cip1) is considered as a negative regulator of the cell cycle and a tumor suppressor. Previously, Kawahara *et al* found that overexpression of p21 correlates with malignant behavior in GEP-NETs patients (15). In the present study, high p21 expression was found in 23/68 (34%) of the 68 patients. However, no prognostic significance for p21 was identified.

In conclusion, the current study demonstrates a prognostic relevance for p16. Low expression of p16 was found to correlate with a shorter overall survival in patients graded as the G2 subgroup. Results of the present study indicate the value of the incorporation of immunohistochemical expression of p16 into a new classification to individualize therapeutic strategies within this subgroup in the future.

## Figures and Tables

**Table I:** Immunostaining results for p16 and p21.

	Cases, n (%)	
	
Expression	p16	p21
Negative	37 (54)	36 (53)
<5%	2 (3)	9 (13)
5–50%	23 (34)	19 (28)
>50%	6 (9)	4 (6)

**Table II:** Patient demographics and clinical features.

		p16 expression			p21 expression		
			
Variable	Total, n	High (%)	Low (%)	P-value^a^	High (%)	Low (%)	P-value^a^
Gender				0.16			0.77
Male	51	26 (51)	25 (49)		18 (35)	33 (65)	
Female	17	5 (29)	12 (71)		5 (29)	12 (71)	
Age, years				0.05			0.61
<60	38	13 (34)	25 (66)		14 (37)	24 (63)	
≥60	30	18 (60)	12 (40)		9 (30)	21 (70)	
Tumor origin				0.12			0.29
Gastric	51	27 (53)	24 (47)		15 (29)	36 (71)	
Colon and rectum	10	2 (20)	8 (80)		4 (40)	6 (60)	
Pancreas	7	2 (29)	5 (71)		4 (57)	3 (43)	
WHO classification				0.12			0.35
G1	9	2 (22)	7 (78)		5 (56)	4 (44)	
G2	37	21 (57)	16 (43)		11 (30)	26 (70)	
G3	22	8 (36)	14 (64)		7 (32)	15 (68)	
Tumor size, cm				0.53			0.16
<2	11	6 (55)	5 (45)		6 (55)	5 (45)	
>2	57	25 (44)	32 (56)		17 (30)	40 (70)	
Functional status				1.00			1.00
Nonfunctional	51	23 (45)	28 (55)		17 (33)	34 (67)	
Functional	17	8 (47)	9 (53)		6 (35)	11 (65)	
Metastasis				0.81			0.08
Negative	28	12 (43)	16 (57)		13 (46)	15 (54)	
Positive	40	19 (47)	21 (53)		10 (25)	30 (75)	
Localization of metastases				0.69			0.06
Liver	8	4 (50)	4 (50)		4 (50)	4 (50)	
Other	27	10 (37)	17 (63)		4 (15)	23 (85)	

a Fisher’s exact test.

**Table III:** RR of succumbing to GEP-NETs.

	Univariate analysis			Multivariate analysis		
		
Variable	RR	95% CI	P-value	RR	95% CI	P-value
Gender			0.13			
Male	1.0	-				
Female	1.7	0.9–3.4				
Age, years			0.20			
<60	1.0	-				
≥60	1.4	0.8–2.4				
Tumor origin			0.05			
Gastric	1.0	-				
Colon and rectum	0.7	0.3–1.5				
Pancreas	0.1	0–0.5				
WHO Classification			<0.01			<0.01
G1	1.0	-		1.0	-	
G2	13.5	1.8–99.1		15.1	1.9–122.5	
G3	25.5	3.4–193.0		26.8	3.4–213.0	
Tumor size, cm			0.08			
<2	1.0	-				
>2	2.2	0.9–5.1				
Functional status			0.84			
Nonfunctional	1.0	-				
Functional	0.9	0.5–1.8				
Metastasis			<0.01			0.01
Negative	1.0	-		1.0	-	
Positive	2.5	1.4–4.4		2.1	1.2–3.7	
Localization of metastases			0.41			
Liver	1.0	-				
Other	0.7	0.3–1.6				
p16 expression			0.25			0.09
High (+)	1.0	-		1.0	-	
Low (−)	1.4	0.8–2.4		1.7	0.9–3.0	
p21 expression			0.16			0.12
Low (<5%)	1.0	-		1.0	-	
High (>5%)	0.7	0.4–1.2		0.6	0.3–1.1	

RR, relative risk; GEP-NETs, gastroenteropancreatic neuroendocrine tumors.

**Table IV:** RR of mortality in the G2 group of GEP-NETs (n=37).

	Univariate analysis		
	
Variable	RR	95% CI	P-value
Gender			0.91
Male	1.0	-	
Female	1.0	0.5–2.3	
Age, years			0.78
<60	1.0	-	
≥60	0.9	0.4–1.8	
Tumor origin			0.63
Gastric	1.0	-	
Colon, rectum and pancreas	0.8	0.3–2.1	
Tumor size, cm			0.48
<2	1.0	-	
>2	1.5	0.5–5.1	
Functional status			0.30
Nonfunctional	1.0	-	
Functional	1.5	0.7–3.1	
Metastasis			0.14
Negative	1.0	-	
Positive	1.8	0.8–3.8	
Localization of the metastases			0.85
Liver	1.0	-	
Other	0.9	0.3–2.4	
p16 expression			<0.01
High (+)	1.0	-	
Low (−)	4.4	1.8–10.6	
p21 expression			0.99
Low (<5%)	1.0	-	
High (>5%)	1.0	0.5–2.2	

RR, relative risk; GEP-NETs, gastroenteropancreatic neuroendocrine tumors.
